# Pilot evaluation of a single oral fecal microbiota transplantation for canine atopic dermatitis

**DOI:** 10.1038/s41598-023-35565-y

**Published:** 2023-05-31

**Authors:** Koji Sugita, Ayaka Shima, Kaho Takahashi, Genki Ishihara, Koji Kawano, Keitaro Ohmori

**Affiliations:** 1 Cooperative Division of Veterinary Sciences, Graduate School of Agriculture, Tokyo University of Agriculture and Technology, Tokyo, Japan; 2 Sugita Animal Hospital, Saitama, Japan; 3 Anicom Specialty Medical Institute Inc., Tokyo, Japan; 4 Tokyo Animal Allergy Center, Tokyo, Japan; 5 Department of Gastroenterology and Gastroenterological Oncology, Fujita Health University, Aichi, Japan; 6 Division of Animal Life Science, Institute of Agriculture, Tokyo University of Agriculture and Technology, Tokyo, Japan

**Keywords:** Preclinical research, Skin diseases

## Abstract

The gut microbiota has been suggested to be involved in the pathogenesis of canine atopic dermatitis (cAD). However, the gut microbiota has not been well characterized in dogs with atopic dermatitis (AD). In addition, the efficacy of fecal microbiota transplantation (FMT) in dogs with AD remains unclear. This research, therefore, aimed to characterize the gut microbiota of dogs with AD and conduct pilot evaluation of the efficacy of a single oral FMT on clinical signs and the gut microbiota of dogs with AD. For these purposes, we used 12 dogs with AD and 20 healthy dogs. The 16S rRNA analysis of the fecal microbiota revealed significant differences between 12 dogs with AD and 20 healthy dogs. Next, a single oral FMT was performed in 12 dogs with AD as a single-arm, open-label clinical trial for 56 days. A single oral FMT significantly decreased Canine Atopic Dermatitis Extent and Severity Index (CADESI)-04 scores from day 0 (median score, 16.5) to day 56 (8) and Pruritus Visual Analog Scale (PVAS) scores from days 0 (median score, 3) to day 56 (1). Furthermore, a single oral FMT changed the composition of the fecal microbiota of dogs with AD at the phylum and genus levels. The number of common amplicon sequence variants in the fecal microbiota between donor dogs and dogs with AD was positively correlated with CADESI-04 and PVAS reduction ratios 56 days after FMT. Our findings suggest that the gut microbiota plays a pivotal role in the pathogenesis of cAD, and that oral FMT could be a new therapeutic approach targeting the gut microbiota in cAD.

## Introduction

Canine atopic dermatitis (cAD) is an allergic inflammatory and pruritic skin disease^1^. The pathogenesis of cAD involves multiple factors, including genetic predisposition, aberrant immune responses to environmental allergens, and skin barrier dysfunction^2,3^. cAD is considered a natural homolog of human atopic dermatitis (AD) because of the similarities in clinical features and the pathogenesis^4^. A variety of treatment options, such as glucocorticoids, cyclosporin, Janus kinase inhibitor, anti-dog interleukin-31 antibody, and allergen-specific immunotherapy, are available for dogs with AD^2,5^. However, it is often difficult to control clinical signs of cAD. To accomplish effective and long-term management of cAD, alternative therapeutic targets based on the pathogenesis need to be explored.

The gut microbiota plays an essential role in maintaining homeostasis in humans and animals, including immunomodulation, physical barrier against intestinal pathogens, provision of nutrients, and regulation of host metabolism^6–8^. Intestinal dysbiosis is a clear shift of the gut microbiota from the normal outside of a healthy population, and has been reported in various gastrointestinal (GI) and extra-GI diseases, including AD, in humans^8^. Next-generation sequencing (NGS) analysis revealed intestinal dysbiosis in dogs with several GI and extra-GI diseases, such as acute diarrhea^9^, inflammatory bowel disease (IBD)^9,10^, heart diseases^11,12^, and obesity^13^. A recent study reported that treatment with a nutraceutical improved the index of intestinal dysbiosis, as assessed by seven bacterial taxa in dogs with AD^14^. However, the gut microbiota has not been characterized in detail by NGS in dogs with AD and has not been compared with healthy dogs. Thus, it remains unclear whether the microbial shift exists in the GI tract of dogs with AD. In addition, the role of the gut microbiota in the development of cAD and as a therapeutic target remains poorly understood.

Fecal microbiota transplantation (FMT) is a treatment approach in which feces from a healthy individual is transplanted into the GI tract of a diseased person^15,16^. Although the detailed mechanisms of FMT have not been fully elucidated, it has been shown that it corrects intestinal dysbiosis by increasing the number and variety of beneficial bacteria and restoring the diversity and function of the gut microbiota^17^. In human medicine, FMT is widely used as an effective treatment for recurrent or refractory *Clostridium difficile* infection (CDI)^18^. Meta-analyses have revealed the clinical efficacy of FMT for human GI and extra-GI diseases other than CDI, including IBD, hepatic disorders, metabolic syndrome, and antibiotic-resistant organisms^19^. Recently, the efficacy and safety of FMT have also been reported in a small population of human AD^20,21^. In dogs, FMT was shown to be effective for several GI diseases, including parvovirus infection^22^, acute diarrhea^23^, *C. difficile*-associated diarrhea^24^, IBD^25–28^, and chronic enteropathy^29^. However, the efficacy of FMT for extra-GI diseases, including cAD, has not been evaluated in dogs. Considering the potential effect of FMT in human AD, FMT could be a promising therapy for cAD.

Therefore, the purposes of this study were to characterize the gut microbiota of 12 dogs with AD using NGS and to perform pilot evaluation of the efficacy of a single oral FMT on clinical signs and the gut microbiota of 12 dogs with AD.

## Results

### Analysis of the fecal microbiota of dogs with AD and healthy dogs

**Figure 1:**
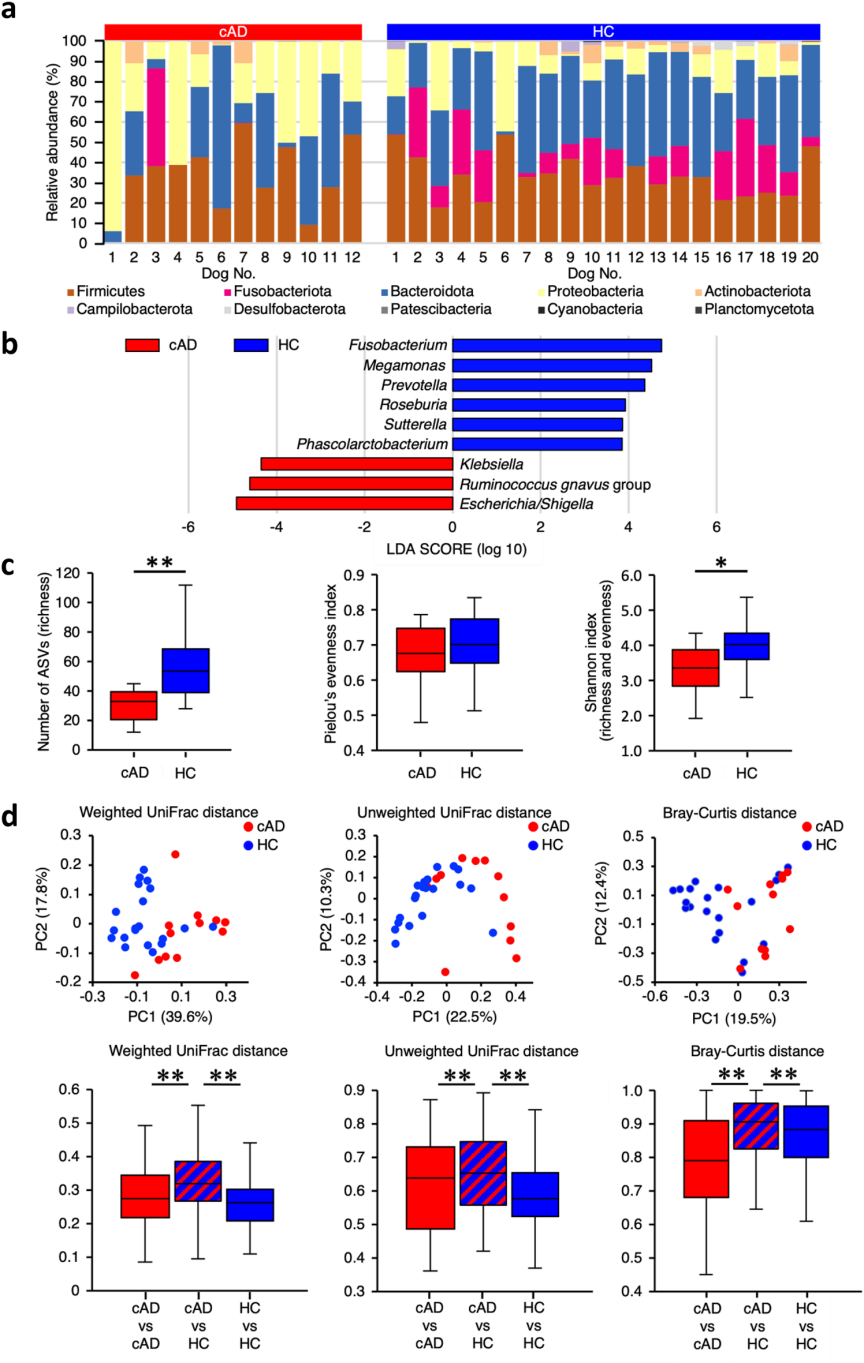
Comparison of the fecal microbiota between dogs with atopic dermatitis (AD) and healthy control (HC) dogs. **(a)** The composition of the fecal microbiota at the phylum level in 12 dogs with AD and 20 HC dogs. **(b)** Linear discriminant analysis effect size (LEfSe) of the fecal microbiota at the genus level in dogs with AD and HC dogs. Data of the fecal microbiota were compared using the Kruskal–Wallis test. Cut-off value: |LDA score|> 3 and P < 0.05. **(c)** Comparison of the alpha diversity of the fecal microbiota in 12 dogs with AD and 20 HC dogs. Data between the two groups were compared using the Mann–Whitney *U* test for the number of ASVs and the unpaired *t* test for the Pielou’s evenness and Shannon index. **(d)** The beta diversity of the fecal microbiota in 12 dogs with AD and 20 HC dogs. Data shows the principal coordinates analysis and box-and-whisker plots of weighted UniFrac, unweighted UniFrac, and Bray–Curtis distances. Data among the three groups were compared using the Permutational Multivariate Analysis of Variance. *P < 0.05, **P < 0.01. *cAD* canine atopic dermatitis.

We first compared the fecal microbiota of 12 dogs with AD (Table 1) with that of age-, sex-, and breed-matched 20 healthy control (HC) dogs (Table 2). The fecal microbiota of dogs with AD at the phylum level was predominantly composed of Firmicutes, Bacteroidota, and Proteobacteria, whereas that of HC dogs mainly comprised Firmicutes, Fusobacteriota, Bacteroidota, Proteobacteria, and Actinobacteriota (Fig. 1a). At the genus level, compared with HC dogs, the fecal microbiota of dogs with AD had significantly lower occupancy of *Fusobacterium*, *Megamonas*, *Prevotella*, *Roseburia*, *Sutterella*, and *Phascolarctobacterium* and higher numbers of *Escherichia/Shigella*, *Ruminococcus gnavus* group, and *Klebsiella* (Fig. 1b, Supplementary Fig. S1). Among the alpha diversity indices of the fecal microbiota, richness of bacteria, shown by the number of observed amplicon sequence variants (ASVs), and Shannon index were significantly lower in dogs with AD than in HC dogs (richness, P = 0.0045; Shannon index, P = 0.0196) (Fig. 1c). Principal coordinates analysis (PCoA) of compositional dissimilarity showed that the fecal microbiota of dogs with AD was plotted separately from that of HC dogs (Fig. 1d). Weighted UniFrac, unweighted UniFrac, and Bray–Curtis distances of the fecal microbiota were significantly different between dogs with AD and HC dogs (Weighted and unweighted: P = 0.001, respectively; Bray–Curtis: P = 0.002) (Fig. 1d). These findings clearly demonstrate the presence of microbial shifts in the dogs with AD included in this study.

**Table 1:** Clinical characteristics of 12 dogs with atopic dermatitis.

Case no.	Breed	Sex	Age (years)	Weight (kg)	Donor	Dose of feces (g/kg)
1	Yorkshire Terrier	Male, castrated	11.3	3.5	A	11.6
2	Shiba	Female, spayed	1.5	8.1	A	2.6
3	French Bulldog	Male, castrated	2.4	11.9	A	1.6
4	Shiba	Female, spayed	14.0	7.9	A	3.6
5	Beagle	Male, castrated	8.1	9.2	A	2.4
6	Mixed breed	Male, castrated	4.4	20.7	A	2.3
7	French Bulldog	Female	5.6	8.0	A	3.0
8	Cavalier King Charles Spaniel	Male, castrated	8.1	10.0	B	2.4
9	Toy poodle	Female, spayed	6.2	3.3	B	3.8
10	Welsh Terrier	Female, spayed	7.1	7.4	B	1.8
11	Lakeland Terrier	Male, castrated	1.7	5.1	B	4.5
12	Yorkshire Terrier	Female	6.0	1.5	B	5.0

**Table 2:** Comparison of clinical characteristics between 12 dogs with atopic dermatitis and 20 healthy control dogs.

Variable	cAD (n = 12)	HC (n = 20)	P value
Median age, years (range)	6.1 (1.5–14.0)	6.4 (1.0–14.3)	0.75
Male, number (%)	6 (50)	11 (55)	1.00
Female, number (%)	6 (50)	9 (45)	1.00
Breed			
Yorkshire Terrier, number (%)	2 (17)	4 (20)	1.00
Shiba, number (%)	2 (17)	4 (20)	1.00
French bulldog, number (%)	2 (17)	4 (20)	1.00
Beagle, number (%)	1 (8)	2 (10)	1.00
Mixed breed, number (%)	1 (8)	2 (10)	1.00
Cavalier King Charles Spaniel, number (%)	1 (8)	2 (10)	1.00
Toy Poodle, number (%)	1 (8)	2 (10)	1.00
Welsh Terrier, number (%)	1 (8)	0 (0)	0.38
Lakeland Terrier, number (%)	1 (8)	0 (0)	0.38

Age between the two groups were compared using the unpaired *t* test. Sex and breed between the two groups were compared using the Fisher’s exact test.

*cAD* canine atopic dermatitis, *HC* healthy control.

### Effect of a single oral FMT on clinical signs

**Figure 2:**
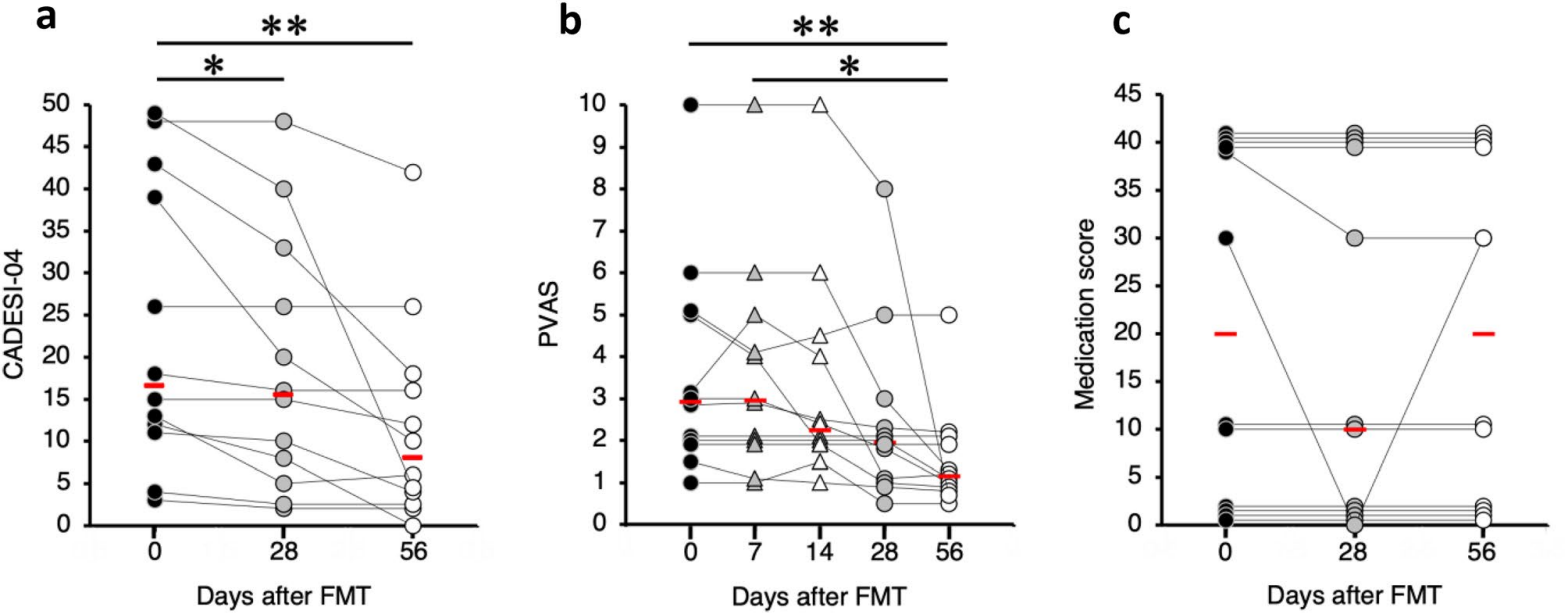
Changes in clinical scores of 12 dogs with atopic dermatitis after a single oral fecal microbiota transplantation. **(a)** Canine Atopic Dermatitis Extent and Severity Index (CADESI)-04. **(b)** Pruritus Visual Analog Scale (PVAS). **(c)** Medication score. The red bars indicate the median in each group. Clinical scores were compared using the Friedman test, followed by the Scheffe’s multiple comparison test. *P < 0.05, **P < 0.01.

Next, we performed a single oral FMT in 12 dogs with AD as a single-arm, open-label trial. A single oral FMT significantly decreased Canine Atopic Dermatitis Extent and Severity Index (CADESI)-04 scores from day 0 (median score, 16.5; range 3–49) to day 28 (median score, 15.5; range 2–48; P = 0.0337) and day 56 (median score, 8; range 0–42; P < 0.001) (Fig. 2a); the reductions were observed in 11 of the 12 dogs with AD (92%) on day 56. A single oral FMT also significantly decreased Pruritus Visual Analog Scale (PVAS) scores from days 0 (median score, 3; range 1–10) and 7 (median score, 3; range 1–10) to day 56 (median score, 1; range 0.5–5, P = 0.0049 and P = 0.0415, respectively) (Fig. 2b); the reductions were detected in 9 of the 12 dogs with AD (75%) on day 56. Medication scores did not change significantly during the study period (P = 0.2231) (Fig. 2c). A decrease in medication scores was found in one of the 12 dogs with AD (8%) on day 56; however, increases in medication scores were not observed in any dog with AD.

### Adverse events

Four of the 12 dogs with AD (nos. 5, 7, 8, and 9) excreted mild soft feces for 1–3 days after oral FMT.

### Effect of a single oral FMT on the fecal microbiota

**Figure 3:**
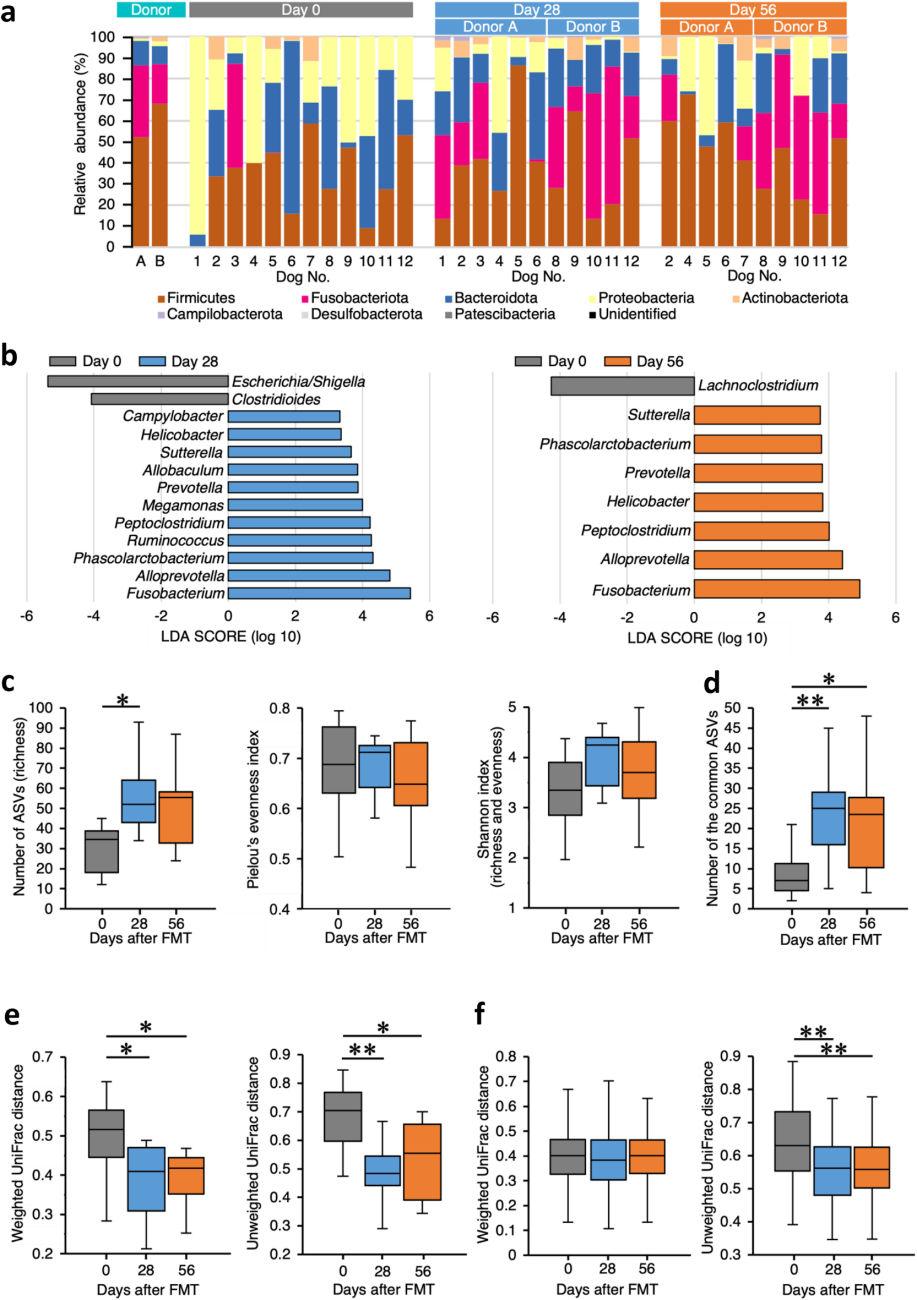
Changes in the fecal microbiota in dogs with atopic dermatitis (AD) before and after a single oral fecal microbiota transplantation (FMT). **(a)** Comparison of the fecal microbiota at the phylum level between the two donor dogs and 12 dogs with AD before (day 0) and 28 and 56 days after a single oral FMT. **(b)** Linear discriminant analysis effect size (LEfSe) of fecal microbiota at the genus level in 12 dogs with AD before (day 0) and 28 and 56 days after a single oral FMT. Data of the fecal microbiota were compared using the Kruskal–Wallis test. Cut-off value: |LDA score|> 3 and P < 0.05. **(c)** The alpha diversity of the fecal microbiota in 12 dogs with AD before (day 0) and 28 and 56 days after a single oral FMT. Data among the three groups were compared using the one-way analysis of variance (ANOVA), followed by the Sheffe’s multiple comparison test. **(d)** The number of the common amplicon sequence variants (ASVs) between two donor dogs and 12 dogs with AD before (day 0) and 28 and 56 days after a single oral FMT. Data among the three groups were compared using the one-way ANOVA, followed by the Sheffe’s multiple comparison test. **(e)** The beta diversity of the fecal microbiota in two donor dogs and 12 dogs with AD before (day 0) and 28 and 56 days after a single oral FMT. Data shows the box-and-whisker plots of weighted and unweighted UniFrac distances. Data among the three groups were compared using the one-way ANOVA, followed by the Sheffe’s multiple comparison test. **(f)** The beta diversity of the fecal microbiota in 20 healthy control dogs and 12 dogs with atopic dermatitis before (day 0) and 28 and 56 days after a single oral FMT. Data shows the box-and-whisker plots of weighted and unweighted UniFrac distances. Data among the three groups were compared using the Kruskal–Wallis test, followed by the Steel–Dwass test. *P < 0.05, **P < 0.01.

A single oral FMT changed the composition of the fecal microbiota in dogs with AD. At the phylum level, Fusobacteriota and Actinobacteriota were noted in the fecal microbiota of dogs with AD after oral FMT (Fig. 3a). At the genus level, the fecal microbiota of dogs with AD before oral FMT was characterized by *Escherichia/Shigella*, *Clostridioides*, and *Lachnoclostridium* compared with that after oral FMT (Fig. 3b). In contrast, the fecal microbiota of dogs with AD was characterized by *Fusobacterium*, *Alloprebotella*, *Phascolarctobacterium*, *Ruminococcus*, *Peptoclostridium*, *Megamonas*, *Prevotella*, *Allobaculum*, *Sutterella*, *Helicobacter*, and *Campylobacter* 28 days after oral FMT (Fig. 3b), and *Fusobacterium*, *Alloprebotella*, *Peptoclostridium*, *Helicobacter*, *Prevotella*, *Phascolarctobacterium*, and *Sutterella* 56 days after oral FMT, compared with that before oral FMT (Fig. 3b). Among the alpha diversity indices of the fecal microbiota, richness was significantly increased 28 days after oral FMT (P = 0.0195) (Fig. 3c). In addition, the number of the common ASVs that were 100% matched sequences between donor dogs and dogs with AD was significantly increased after oral FMT (day 28, P = 0.0083; day 56, P = 0.0227) (Fig. 3d). As the beta diversity, weighted and unweighted UniFrac distances between donor dogs and dogs with AD were significantly decreased after oral FMT (weighted: day 28, P = 0.0139; day 56, P = 0.0324; unweighted: day 28, P = 0.0036; day 56, P = 0.0328) (Fig. 3e; PCoA, Supplementary Fig. S2a). When weighted and unweighted UniFrac distances between HC dogs and dogs with AD were compared, the unweighted UniFrac distance significantly decreased after oral FMT (day 28 and 56, P < 0.001, respectively) (Fig. 3f; PCoA, Supplementary Fig. S2b).

### Association between changes in the fecal microbiota and reduction in clinical scores

**Figure 4:**
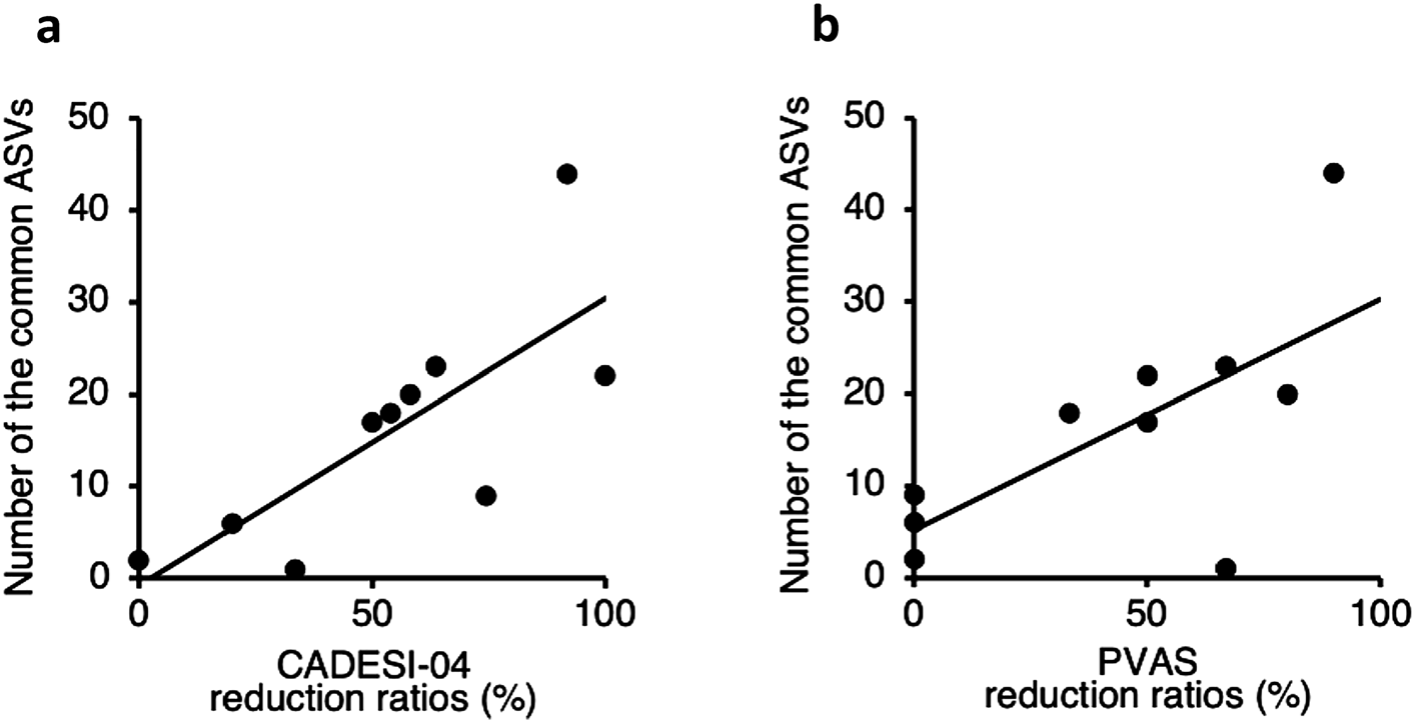
Correlations between the number of the common amplicon sequence variants (ASVs) and reduction ratios in clinical scores in dogs with atopic dermatitis 56 days after a single oral fecal microbiota transplantation. (**a**) Correlations between the number of the common ASVs and Canine Atopic Dermatitis Extent and Severity Index (CADESI)-04 reduction ratios. (**b**) Correlations between the number of the common ASVs and Pruritus Visual Analog Scale (PVAS) reduction ratios. The correlations were evaluated using the Pearson’s rank correlation coefficient.

In the fecal microbiota of dogs with AD, the number of the common ASVs was positively correlated with CADESI-04 and PVAS reduction ratios 56 days after oral FMT (CADESI-04: P = 0.0113, *r* = 0.7564; PVAS: P = 0.0320, *r* = 0.6755) (Fig. 4a,b). Taxonomic analysis of the common ASVs revealed that thirty-six bacteria were found to be significantly correlated with reduction in clinical scores (Supplementary Table S1–S4). At the genus level, 14 and 12 genera were positively correlated with the CADESI-04 and PVAS reduction ratios, respectively (P < 0.05, *ρ* > 0.4) (Table 3). Furthermore, five and seven genera were negatively correlated with the CADESI-04 and PVAS scores after oral FMT, respectively (P < 0.05, *ρ* <  − 0.4) (Table 3).

**Table 3:** Bacterial genera that were significantly correlated with reduction in clinical scores.

Genus	CADESI-04 reduction ratio	PVAS reduction ratio	CADESI-04	PVAS
*Fusobacterium*	+	+	+	+
*Sutterella*	+	+	+	+
*Romboutsia*	+	+	+	−
*Phascolarctobacterium*	+	+	−	+
*Bacteroides*	+	+	−	−
*Intestinimonas*	+	+	−	−
*Megamonas*	+	+	−	−
*Peptoclostridium*	+	+	−	−
*Helicobacter*	+	−	−	+
*Allobaculum*	+	−	−	−
*Alloprevotella*	+	−	−	−
*Butyricicoccus*	+	−	−	−
*Catenibacterium*	+	−	−	−
*Prevotella*	+	−	−	−
*Blautia*	−	+	+	+
*Lachnospiraceae* NK4A136 group	−	+	+	−
*Erysipelatoclostridium*	−	+	−	+
*Collinsella*	−	+	−	−
*Holdemanella*	−	−	−	+

In the fecal microbiota of dogs with AD, bacterial taxonomy of the common ASVs that were significantly correlated with reduction in clinical scores were summarized at the genus level.

The correlations between bacteria (genus level) and reduction ratios in clinical scores and those between bacteria (genus level) and clinical scores after oral FMT were evaluated using the Spearman’s rank correlation coefficient.

*CADESI-04* canine atopic dermatitis extent and severity index, *PVAS* pruritus visual analog scale.

+, significant correlations (positive correlation, P < 0.05 and *ρ* > 0.4; negative correlation, P < 0.05 and *ρ* <  − 0.4).

−, no significant correlations (P > 0.05).

## Discussion

The present study demonstrated the significant differences in the fecal microbiota between dogs with AD and healthy dogs. Furthermore, a single oral FMT significantly decreased skin lesions and pruritus scores, and changed the fecal microbiota in dogs with AD. These findings suggest that the gut microbiota plays a pivotal role in the pathogenesis of cAD, and that oral FMT could be a new therapeutic approach targeting the gut microbiota in cAD.

In this study, we found Firmicutes, Fusobacteriota, Bacteroidota, Proteobacteria, and Actinobacteriota to be the major components of the fecal microbiota of healthy dogs, similar to a previous study^30^. In contrast, Firmicutes, Bacteroidota, and Proteobacteria occupied the fecal microbiota of dogs with AD. We also showed that Fusobacteriota and Actinobacteriota increased in the fecal microbiota of dogs with AD after oral FMT. Therefore, the lower abundance of Fusobacteriota and Actinobacteriota may be characteristic of the fecal microbiota of cAD at the phylum level. It was also seen that as compared to HC dogs, the fecal microbiota of dogs with AD had lower occupancy of *Fusobacterium*, *Megamonas*, *Prevotella*, *Roseburia*, *Sutterella*, and *Phascolarctobacterium*, and higher numbers of *Escherichia/Shigella*, *Ruminococcus gnavus* group, and *Klebsiella*. These may be a feature of the fecal microbiota of cAD at the genus level. However, similar microbial shifts were reported in dogs with GI diseases or after antibiotic use^31^. Thus, to characterize the cAD-specific gut microbiota, further analysis would be required using a large population of healthy dogs, AD dogs, dogs with GI diseases, and dogs on broad-spectrum antibiotics.

The mechanisms underlying FMT are not fully understood. However, it may be associated with increased microbial diversity, restoration of normal microbiota, and enhanced numbers of beneficial bacteria^17^. In this study, a single oral FMT significantly decreased CADESI-04 and PVAS scores without changing the medication scores in dogs with AD. It also significantly increased bacterial diversity, as assessed by the number of ASVs (richness). In particular, the number of the common ASVs between donor dogs and dogs with AD was noted to have significantly increased after a single oral FMT. Furthermore, positive correlations between the number of the common ASVs and CADESI-04 and PVAS reduction ratios were detected in dogs with AD. These results suggest that oral FMT may have introduced a large number of donor-derived bacteria into the GI tract of dogs with AD, thereby decreasing clinical scores associated with cAD. Alternatively, oral FMT may have stimulated the gut microbiota of dogs with AD to increase the microbial diversity. We also demonstrated that among the common ASVs increased after oral FMT, 19 bacterial genera were significantly correlated with reduction in clinical scores in dogs with AD. In particular, *Fusobacterium* and *Sutterella* were positively correlated with CADESI-04 and PVAS reduction ratios and were negatively correlated with CADESI-04 and PVAS scores. Thus, among the 19 bacterial genera, *Fusobacterium* and *Sutterella* might be more crucial toward the reduction in clinical scores associated with cAD. To further determine the importance of these bacterial genera as contributors to the efficacy of oral FMT in cAD, detailed investigations are required.

Guidelines for FMT have been proposed in dogs^32,33^. However, there is no established method or route of fecal administration in dogs. Previous studies conducted FMT in dogs via rectal enema^22,23,26,27^, endoscopic introduction into the small or large intestine^25,28^, oral administration of fecal solution^24^ or frozen feces^25^, and oral capsules of concentrated lyophilized feces^29^. Based on our previous report^24^, in this study, we selected oral administration of fecal solution for dogs with AD. As a result, a single oral FMT significantly decreased clinical scores and changed the gut microbiota in dogs with AD. Our findings thus suggest that even a single oral FMT may be an effective method for treating cAD for at least 56 days. To determine the long-term effects of a single oral FMT on clinical sings and the gut microbiota of dogs with AD, a longer follow-up period for more than 56 days would be needed.

Although FMT has been considered a safe treatment, a recent study reported bacteremia due to drug-resistant *Escherichia coli* occurred in two human patients after FMT, and one died due to this severe infection^34^. Thus, safety is an important issue in FMT. In this study, although four of the 12 dogs with AD briefly excreted mild soft feces after oral FMT, no severe adverse events were observed. In addition, we previously reported that oral FMT did not induce any adverse events in a dog with *C. difficile*-associated diarrhea^24^. These results suggest that oral FMT might be a safe and well-tolerated treatment for dogs. However, this study was designed as an uncontrolled study. Therefore, to evaluate the safety of oral FMT for cAD, the frequency and severity of adverse events associated with oral FMT should be compared between the study and control groups in future studies.

The dogs with AD in this study were categorized as cAD in remission, mild, or moderate cAD according to the CADESI-04^35^ and had already received other therapies, excluding antibiotics, against cAD. Therefore, we could not assess the clinical efficacy of a single oral FMT for severe cAD or untreated dogs with AD. Oral administration of fecal solution is a simple procedure that does not require anesthesia, sedation, endoscopic equipment, or specialized capsule, and can be repeated easily. Thus, repeated oral FMT may induce favorable outcomes in dogs with severe AD or untreated dogs with AD.

In this study, antibiotics had not been administered to the dogs with AD for 2 months prior to the study enrollment and during the study period. However, the histories of antibiotic use in the dogs with AD before more than 2 months prior to the study enrollment could not be traced. Thus, we can’t rule out the possibility that previous antibiotic use might have affected the gut microbiota in the dogs with AD.

In conclusion, the present study revealed that a single oral FMT significantly decreased skin lesions and pruritus scores and changed the gut microbiota in dogs with AD. Since this study was designed as a pilot trial with a short observation period (56 days), further studies are needed to clarify the long-term effect of a single or repeated oral FMT on cAD using a large population of dogs with mild to severe AD and appropriate controls. Nevertheless, this study provides evidence for a crucial role of the gut microbiota in the pathogenesis and a therapeutic target of cAD.

## Materials and methods

### Dogs

Twelve dogs diagnosed with AD according to established clinical criteria^36^ were enrolled in this study (Table 1). Eight of the 12 dogs had already received other therapies (Table 4), but none of the 12 dogs were administered antibiotics for two months prior to the study inclusion and during the study period. To compare the fecal microbiota of dogs with AD, age-, sex-, and breed-matched 20 healthy dogs were used as a healthy control (HC) group (Table 2). Two healthy dogs (donor A: an 11-year-old, intact male beagle weighing 12.4 kg; donor B: a 5-year-old, intact male beagle weighing 11.3 kg) maintained for research purposes at Tokyo University of Agriculture and Technology were used as donor dogs for oral FMT. The donor dogs were fed a commercial diet (Science Diet Adult, Hill’s-Colgate Ltd., Tokyo, Japan), and water was provided ad libitum. The donor dogs showed no clinical signs. Physical and clinical examinations, including a complete blood count, serum biochemical analysis, radiography, abdominal ultrasound, and fecal examination, did not identify any abnormalities in the donor dogs. The fecal samples of the donor dogs were subjected to IDEXX Canine Diarrhea real-time PCR Panels analysis (IDEXX Laboratories, Inc., Tokyo, Japan) and were found to be negative for *Cryptosporidium* spp., *Giardia* spp., *Clostridium perfringens* α toxin, *C. difficile* toxin A&B, *Campylobacter jejuni*, *Campylobacter coli*, Salmonella spp., canine parvovirus type 2, canine distemper virus, and canine enteric coronavirus genes.

**Table 4:** Medication scores.

Medication	Score
No concurrent medication	0
Shampoo therapy	5
Ear medication (topical)	5
Other topical therapy	5
Antihistamies	10
Prednisolone	
≥ 1 mg/kg/day	40
0.5–1 mg/kg/day	30
0.2–0.5 mg/kg/day	20
≤ 0.2 mg/kg/day	10
Cyclosporin (5 mg/kg)	
Once daily	30
Every other day	20
Every 3 days	10
Every 4 days	5
Oclacitinib (0.4–0.6 mg/kg)	
Twice daily	40
Once daily	30
Every other day	20
Every 3 days	10
Recombinant Der f 2 immunotherapy 1shot	10

*Der f 2* *Dermatophagoides farina* 2.

### Oral FMT

A single oral FMT was designed as a single-arm, open-label clinical trial. The study period lasted 56 days. Oral FMT was performed according to a procedure reported previously^24^. In brief, immediately after collection of naturally excreted feces from the donor dogs, they were mixed with tap water. The fecal solution was filtered twice through a medical gauze pad, and then administered orally to dogs with AD using a syringe. The average fecal weight administered was 4 g/kg (range 2–12 g/kg). The average total fecal solution administered was 26 mL/dog (range 15–50 mL/dog). Skin lesions, pruritus, medication scores, and fecal microbiota were evaluated in 12 dogs with AD at study inclusion and after oral FMT.

### Evaluation of skin lesions

Skin lesions of dogs with AD were scored using the CADESI-04^35^ by the same veterinarian at study inclusion (day 0) and 28 and 56 days after oral FMT. The CADESI-04 assessed three lesions (erythema, lichenification, and alopecia/excoriation) with four severity scores (0, none; 1, mild; 2, moderate; 3, severe) at 20 body sites, giving a maximum score of 20 × 3 × 3 = 180. According to the CADESI-4, disease severity of cAD is categorized as follows: < 10, normal dogs or cAD in remission; 10–34, mild cAD; 35–59, moderate cAD; ≥ 60, severe cAD^35^.

### Evaluation of pruritus

The severity of pruritus was scored from 0 (no pruritus) to 10 (extremely severe pruritus) using the PVAS^37^ by the owners of dogs with AD at study inclusion (day 0) and 14, 28, and 56 days after oral FMT.

### Evaluation of medication scores

The medications administered to dogs with AD were scored as reported previously^38^, with slight adaptation for this study by inclusion of an additional medication [a recombinant *Dermatophagoides farinae* 2 immunotherapy shot (Allermmune®, ZENOAQ; Tokyo, Japan)] (Table 4). Medication scores were assessed by the same veterinarian at study inclusion (day 0) and 28 and 56 days after oral FMT.

### Analysis of the fecal microbiota

Fecal samples (0.1–0.5 g) were collected from 20 HC dogs and 12 dogs with AD before (day 0) and 28 and 56 days after oral FMT. Fecal samples were suspended in 50 mL of phosphate buffered saline (PBS), filtered using a pluriStrainer 100 μm (pluriSelect Life Science, Leipzig, Germany), and centrifuged at 9000×*g* at 4 °C for 10 min. The pellets were washed with 35 mL of PBS and resuspended in PBS at a final concentration of 0.5 g/mL of the initial fecal weight. A 400 μL volume of suspension was subjected to genomic DNA extraction using a Chemagic DNA Stool 200 Kit (PerkinElmer, Waltham, MA, USA). The V3–V4 regions of the 16S rRNA gene were amplified by PCR and subjected to paired-end sequencing using Illumina MiSeq (Illumina, CA, USA)^39,40^. The sequence data were processed using QIIME 2 version 2020.6^41^. The DADA2 software package version 2020.2.0 incorporated into QIIME 2 was used to correct the amplicon sequence errors and yield high quality reads^42^. To rarefy the data, we used 5000 high quality reads from each sample. Unique ASVs of the 16S rRNA gene and its abundances were summarized in an ASV abundance table, and the alpha and beta diversities were calculated using QIIME2. Microbial taxonomy was assigned using a Naïve Bayes classifier trained on the SILVA 138 database^43,44^.

### Statistical analysis

The normality of the data was analyzed using the Shapiro–Wilk test. The age difference between dogs with AD and HC dogs was compared using the unpaired *t* test. Other categorical variables were compared using Fisher’s exact test. Clinical scores such as CADESI-04, PVAS, and medication scores were compared using the Friedman test, followed by the Scheffe’s multiple comparison test. The alpha diversity of the fecal microbiota was analyzed using the Mann–Whitney *U* test or the unpaired *t* test, depending the normality of the data; the beta diversity was compared using the Permutational Multivariate Analysis of Variance (PERMANOVA). Other fecal microbiota data were compared using the one-way analysis of variance (ANOVA), followed by the Scheffe’s multiple comparison test, or the Kruskal–Wallis test, followed by the Steel–Dwass test. The correlations between two variables were evaluated using the Spearman’s rank correlation coefficient or the Pearson's correlation coefficient, depending on the normality of the variables. Statistical analyses were performed using BellCurve for Excel (Social Survey Research Information Co., Ltd., Tokyo, Japan) software and QIIME 2 version 2020.6. P < 0.05 was considered statistically significant. To determine different taxa between two groups, the linear discriminate analysis (LDA) effect size (LEfSe) on the Galaxy browser was used^45^. In LEfSe, cut-off value was set at |LDA score|> 3 and P < 0.05.

### Ethical approval

The collection and use of feces from healthy donor dogs were approved by the Institutional Animal Care and Use Committee of Tokyo University of Agriculture and Technology (No. 30-131). Oral FMT was approved by the Research Ethics Committee of Tokyo University of Agriculture and Technology (No. 0016013). Written informed consent was obtained from the owners of the dogs with AD and HC dogs. All experiments were conducted in compliance with the ARRIVE guidelines and in accordance with the relevant guidelines and regulations of Tokyo University of Agriculture and Technology.

## Supplementary Information

Supplementary Information.

## Data Availability

The data have been deposited with links to BioProject accession number PRJDB12998 in the DDBJ BioProject database.
