# Supplementary material for: Pilot evaluation of a single oral fecal microbiota transplantation for canine atopic dermatitis
Source: Sci Rep. 2023 May 31;13:8824. doi: 10.1038/s41598-023-35565-y (PMC10230478; doi:10.1038/s41598-023-35565-y)
Supplement: Supplementary file 1 — Supplementary Information. [file 41598_2023_35565_MOESM1_ESM.pdf]

ASV no.	Taxonomy						P value	ρ
	**Phylum**	**Class**	**Order**	**Family**	**Genus**	**Species**		
The common ASVs between donor A and recipient dogs with AD								
ASV 001	Bacteroidota	Bacteroidia	Bacteroidales	Prevotellaceae	*Alloprevotella*		0.0009	0.71
ASV 002	Firmicutes	Bacilli	Erysipelotrichales	Erysipelatoclostridiaceae	*Catenibacterium*	*Uncultured bacterium*	0.0139	0.57
ASV 003	Firmicutes	Clostridia	Peptostreptococcales-Tissierellales	Peptostreptococcaceae	*Peptoclostridium*	*Uncultured bacterium*	0.0173	0.55
ASV 004	Fusobacteriota	Fusobacteriia	Fusobacteriales	Fusobacteriaceae	*Fusobacterium*	*Gut metagenome*	0.0183	0.55
ASV 005	Proteobacteria	Gammaproteobacteria	Burkholderiales	Sutterellaceae	*Sutterella*		0.0197	0.54
ASV 006	Firmicutes	Clostridia	Peptostreptococcales-Tissierellales	Peptostreptococcaceae	*Romboutsia*		0.0199	0.54
ASV 007	Bacteroidota	Bacteroidia	Bacteroidales	Prevotellaceae	*Prevotella*		0.0206	0.54
ASV 008	Firmicutes	Clostridia	Oscillospirales	Butyricicoccaceae	*Butyricicoccus*	*Butyricicoccus pullicaecorum 1.2*	0.0249	0.53
ASV 009	Bacteroidota	Bacteroidia	Bacteroidales	Bacteroidaceae	*Bacteroides*		0.0289	0.51
ASV 010	Firmicutes	Bacilli	Erysipelotrichales	Erysipelotrichaceae	*Allobaculum*	*Allobaculum stercoricanis DSM 13633*	0.0345	0.50
ASV 011	Bacteroidota	Bacteroidia	Bacteroidales	Bacteroidaceae	*Bacteroides*	*Gut metagenome*	0.0410	0.49
ASV 012	Bacteroidota	Bacteroidia	Bacteroidales	Bacteroidaceae	*Bacteroides*	*Uncultured bacterium*	0.0423	0.48
The common ASVs between donor B and recipient dogs with AD								
ASV 013	Firmicutes	Negativicutes	Acidaminococcales	Acidaminococcaceae	*Phascolarctobacterium*	*Phascolarctobacterium sp. canine oral taxon 149*	0.0023	0.72
ASV 014	Proteobacteria	Gammaproteobacteria	Burkholderiales	Sutterellaceae	*Sutterella*	*Uncultured bacterium*	0.0213	0.59
ASV 015	Fusobacteriota	Fusobacteriia	Fusobacteriales	Fusobacteriaceae	*Fusobacterium*	*Fusobacterium mortiferum*	0.0335	0.55
ASV 016	Firmicutes	Negativicutes	Veillonellales-Selenomonadales	Selenomonadaceae	*Megamonas*	*Uncultured organism*	0.0344	0.55
ASV 017	Campilobacterota	Campylobacteria	Campylobacterales	Helicobacteraceae	*Helicobacter*	*Helicobacter canis*	0.0353	0.55
ASV 003	Firmicutes	Clostridia	Peptostreptococcales-Tissierellales	Peptostreptococcaceae	*Peptoclostridium*	*Uncultured bacterium*	0.0384	0.54
ASV 018	Fusobacteriota	Fusobacteriia	Fusobacteriales	Fusobacteriaceae	*Fusobacterium*		0.0416	0.53
ASV 019	Firmicutes	Clostridia	Oscillospirales	Oscillospiraceae	*Intestinimonas*		0.0451	0.52

Supplementary Table S1. Taxonomy of the common amplicon sequence variants (ASVs) that were significantly correlated with Canine Atopic Dermatitis Extent and Severity Index (CADESI)-04 reduction ratio in dogs with atopic dermatitis (AD). The common ASVs were defined as 100% matched sequences between donor dogs and dogs with AD after a single oral fecal microbiota transplantation. The correlations between ASVs and CADESI-04 reduction ratio were evaluated using the Spearman's rank correlation coefficient. ρ indicates the correlation coefficient.

ASV no.	Taxonomy						P value	ρ
	**Phylum**	**Class**	**Order**	**Family**	**Genus**	**Species**		
The common ASVs between donor A and recipient dogs with AD								
ASV 020	Bacteroidota	Bacteroidia	Bacteroidales	Bacteroidaceae	*Bacteroides*	*Bacteroides plebeius*	0.0025	0.67
ASV 021	Fusobacteriota	Fusobacteriia	Fusobacteriales	Fusobacteriaceae	*Fusobacterium*	*Fusobacterium mortiferum*	0.0108	0.58
ASV 022	Fusobacteriota	Fusobacteriia	Fusobacteriales	Fusobacteriaceae	*Fusobacterium*		0.0108	0.58
ASV 006	Firmicutes	Clostridia	Peptostreptococcales-Tissierellales	Peptostreptococcaceae	*Romboutsia*		0.0372	0.49
The common ASVs between donor B and recipient dogs with AD								
ASV 013	Firmicutes	Negativicutes	Acidaminococcales	Acidaminococcaceae	*Phascolarctobacterium*	*Phascolarctobacterium sp. canine oral taxon 149*	0.0011	0.76
ASV 023	Actinobacteriota	Coriobacteriia	Coriobacteriales	Coriobacteriaceae	*Collinsella*		0.0034	0.70
ASV 003	Firmicutes	Clostridia	Peptostreptococcales-Tissierellales	Peptostreptococcaceae;	*Peptoclostridium*	*Uncultured bacterium*	0.0037	0.70
ASV 024	Firmicutes	Clostridia	Oscillospirales	Ruminococcaceae	*Uncultured*	*Human gut metagenome*	0.0037	0.70
ASV 025	Firmicutes	Bacilli	Erysipelotrichales	Erysipelatoclostridiaceae	*Erysipelatoclostridium*	*Uncultured organism*	0.0112	0.63
ASV 026	Firmicutes	Clostridia	Lachnospirales	Lachnospiraceae	*Lachnospiraceae NK4A136 group*	*Uncultured organism*	0.0116	0.63
ASV 027	Firmicutes	Clostridia	Lachnospirales	Lachnospiraceae	*Blautia*		0.0152	0.61
ASV 019	Firmicutes	Clostridia	Oscillospirales	Oscillospiraceae	*Intestinimonas*		0.0170	0.60
ASV 016	Firmicutes	Negativicutes	Veillonellales-Selenomonadales	Selenomonadaceae	*Megamonas*	*Uncultured organism*	0.0222	0.58
ASV 028	Firmicutes	Bacilli	Erysipelotrichales	Erysipelatoclostridiaceae	*Erysipelatoclostridium*		0.0248	0.58
ASV 018	Fusobacteriota	Fusobacteriia	Fusobacteriales	Fusobacteriaceae	*Fusobacterium*		0.0314	0.56
ASV 029	Firmicutes	Clostridia	Lachnospirales	Lachnospiraceae	*Blautia*	*Blautia glucerasea*	0.0357	0.54
ASV 012	Bacteroidota	Bacteroidia	Bacteroidales	Bacteroidaceae	*Bacteroides*	*Uncultured bacterium*	0.0370	0.54
ASV 014	Proteobacteria	Gammaproteobacteria	Burkholderiales	Sutterellaceae	*Sutterella*	*Uncultured bacterium*	0.0403	0.53
ASV 030	Firmicutes	Clostridia	Lachnospirales	Lachnospiraceae			0.0484	0.52

Supplementary Table S2. Taxonomy of the common amplicon sequence variants (ASVs) that were significantly correlated with Pruritus Visual Analog Scale (PVAS) reduction ratio in dogs with atopic dermatitis (AD). The common ASVs were defined as 100% matched sequences between donor dogs and dogs with AD after a single oral fecal microbiota transplantation. The correlations between ASVs and PVAS reduction ratio were evaluated using the Spearman's rank correlation coefficient. ρ indicates the correlation coefficient.

ASV no.	Taxonomy						P value	ρ
	**Phylum**	**Class**	**Order**	**Family**	**Genus**	**Species**		
The common ASVs between donor A and recipient dogs with AD								
ASV 027	Firmicutes	Clostridia	Lachnospirales	Lachnospiraceae	*Blautia*		0.0007	−0.72
ASV 031	Firmicutes	Clostridia	Lachnospirales	Lachnospiraceae	*Blautia*		0.0061	−0.62
ASV 006	Firmicutes	Clostridia	Peptostreptococcales-Tissierellales	Peptostreptococcaceae	*Romboutsia*		0.0100	−0.59
ASV 026	Firmicutes	Clostridia	Lachnospirales	Lachnospiraceae	*Lachnospiraceae NK4A136 group*	*Uncultured organism*	0.0175	−0.55
ASV 032	Firmicutes	Clostridia	Lachnospirales	Lachnospiraceae			0.0223	−0.53
The common ASVs between donor B and recipient dogs with AD								
ASV 014	Proteobacteria	Gammaproteobacteria	Burkholderiales	Sutterellaceae	*Sutterella*	*Uncultured bacterium*	0.0088	−0.65
ASV 033	Fusobacteriota	Fusobacteriia	Fusobacteriales	Fusobacteriaceae	*Fusobacterium*	*Gut metagenome*	0.0281	−0.57

Supplementary Table S3. Taxonomy of the common amplicon sequence variants (ASVs) that were significantly correlated with Canine Atopic Dermatitis Extent and Severity Index (CADESI)-04 score in dogs with atopic dermatitis (AD). The common ASVs were defined as 100% matched sequences between donor dogs and dogs with AD after a single oral fecal microbiota transplantation. The correlations between ASVs and CADESI-04 score were evaluated using the Spearman's rank correlation coefficient. ρ indicates the correlation coefficient.

ASV no.	Taxonomy						P value	ρ
	**Phylum**	**Class**	**Order**	**Family**	**Genus**	**Species**		
The common ASVs between donor A and recipient dogs with AD								
ASV 034	Firmicutes	Bacilli	Erysipelotrichales	Erysipelotrichaceae	*Uncultured*	*Uncultured bacterium*	0.0127	−0.57
ASV 035	Firmicutes	Bacilli	Erysipelotrichales	Erysipelotrichaceae	*Holdemanella*	*Uncultured bacterium*	0.0127	−0.57
ASV 013	Firmicutes	Negativicutes	Acidaminococcales	Acidaminococcaceae	*Phascolarctobacterium*	*Phascolarctobacterium sp. canine oral taxon 149*	0.0127	−0.57
ASV 017	Campilobacterota	Campylobacteria	Campylobacterales	Helicobacteraceae	*Helicobacter*	*Helicobacter canis*	0.0127	−0.57
ASV 027	Firmicutes	Clostridia	Lachnospirales	Lachnospiraceae	*Blautia*		0.0403	−0.49
ASV 005	Proteobacteria	Gammaproteobacteria	Burkholderiales	Sutterellaceae	*Sutterella*		0.0424	−0.48
The common ASVs between donor B and recipient dogs with AD								
ASV 018	Fusobacteriota	Fusobacteriia	Fusobacteriales	Fusobacteriaceae	*Fusobacterium*		0.0131	−0.62
ASV 036	Fusobacteriota	Fusobacteriia	Fusobacteriales	Fusobacteriaceae	*Fusobacterium*	*Fusobacterium mortiferum*	0.0425	−0.53
ASV 025	Firmicutes	Bacilli	Erysipelotrichales	Erysipelatoclostridiaceae	*Erysipelatoclostridium*	*Uncultured organism*	0.0489	−0.52

Supplementary Table S4. Taxonomy of the common amplicon sequence variants (ASVs) that were significantly correlated with Pruritus Visual Analog Scale (PVAS) score in dogs with atopic dermatitis (AD). The common ASVs were defined as 100% matched sequences between donor dogs and dogs with AD after a single oral fecal microbiota transplantation. The correlations between ASVs and PVAS score were evaluated using the Spearman's rank correlation coefficient. ρ indicates the correlation coefficient.

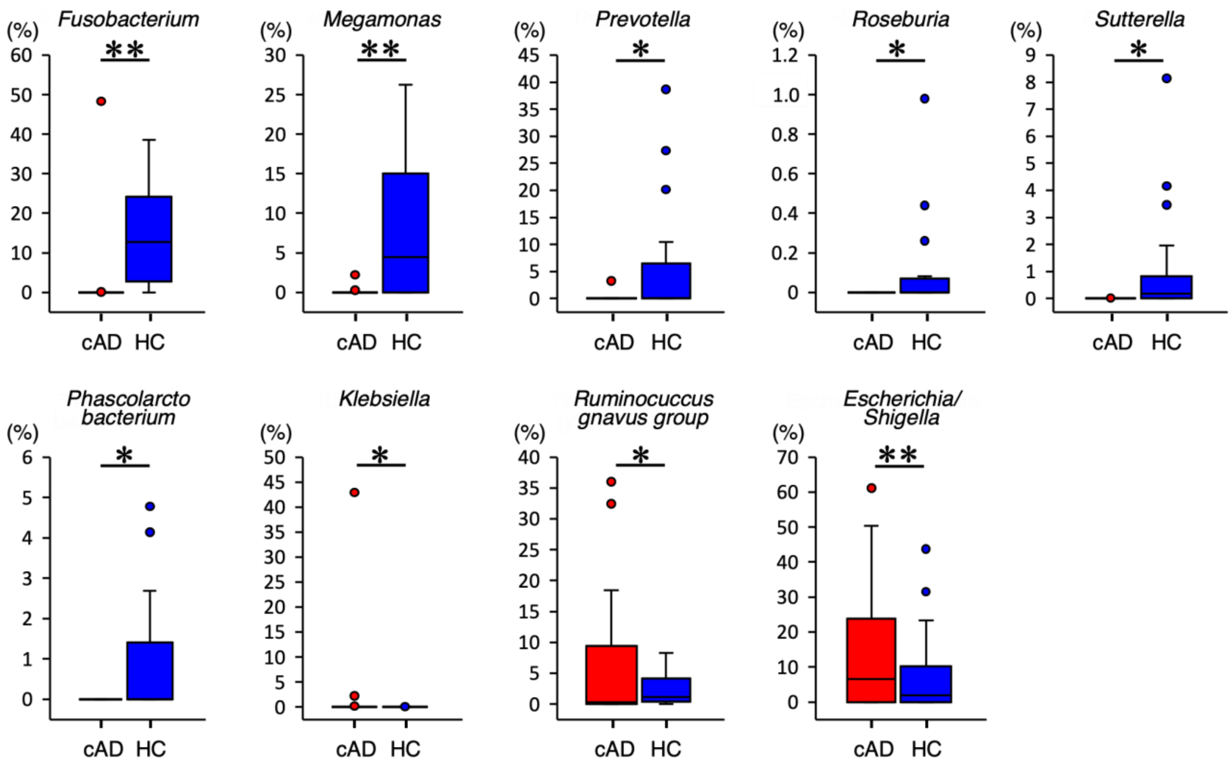

Supplementary Figure S1. Comparison of the fecal microbiota at the genus level between dogs with atopic dermatitis and healthy control dogs. Data shows the percentage of each genus in the fecal microbiota. Data between the two groups were compared using the Mann-Whitney U test. *P < 0.05, **P < 0.01. cAD, canine atopic dermatitis; HC, healthy control.

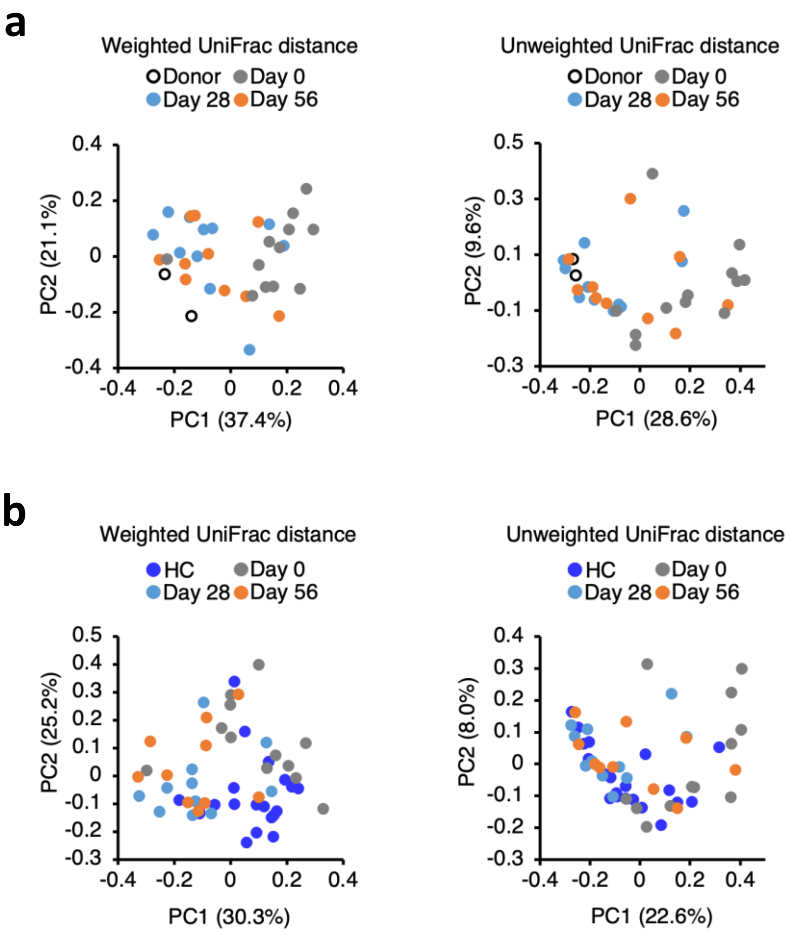

Supplementary Figure S2. The beta diversity of the fecal microbiota in 12 dogs with atopic dermatitis before (day 0) and 28 and 56 days after a single oral FMT and two donor dogs (a) or 20 healthy control dogs (b). Data shows the principal coordinates analysis of weighted and unweighted UniFrac distances. HC, healthy control.
